# American Association of Medical Editors

**Published:** 1870-07

**Authors:** 


### *American Association of Medical Editors.*

We are indebted to the editor of the "Chicago Medical Examiner," Professor N. S. Davis, for advance sheets containing full report of the proceedings of the meeting in Washington, May 2nd, 1870. The American Medical Press was thoroughly represented, and a large number of the editors of Journals were present and signed the articles of association. The following officers were elected for the ensuing year:—

Dr. H. R. Storer, of Boston, President.
Dr. Theophilus Parvin, Vice-President.
Dr. Howard, of Baltimore, Secretary.

The president, Dr. N. S. Davis, of Chicago, then read an address, on "The History, Constitution, and means of Improvement of Medical Journalism in the United States." This address is full of suggestion and instruction, and shows the author fully conversant with our Periodical Medical Literature, its faults and its means of improvement. The paragraph containing this brief history of all our Journals is exceedingly interesting, as is also the whole address. We regret that we are unable to publish it in full.

